# Orofacial Pain with Cardiac Origin of Coronary Artery Disease: A Case Report and Literature Review

**DOI:** 10.1155/2023/6304637

**Published:** 2023-07-12

**Authors:** Eunhye Choi, Yeon-Hee Lee, Hee-Kyung Park

**Affiliations:** ^1^Dental Research Institute, Seoul National University School of Dentistry, Seoul 03080, Republic of Korea; ^2^Department of Oral Medicine and Oral Diagnosis, Bucheon Apple Tree Dental Hospital, 20, Bucheon-ro, Bucheon-si, Gyeonggi-do, Republic of Korea; ^3^Department of Orofacial Pain and Oral Medicine, Kyung Hee University Dental Hospital, Kyung Hee University School of Dentistry, #613 Hoegi-dong, Dongdaemun-gu, Seoul 02447, Republic of Korea; ^4^Department of Oral Medicine and Oral Diagnosis, Dental Research Institute, Seoul National University School of Dentistry, Seoul 03080, Republic of Korea

## Abstract

When diagnosing orofacial pain, clinicians should also consider non-odontogenic origin and systemic diseases as possible etiological factors, along with odontogenic origin. This case report aimed to provide information for early detection of orofacial pain of cardiac origin by dentists, when pain due to coronary artery disease is the only presenting symptom. A 60-year-old male patient with unexplained isolated bilateral jaw pain that had persisted for the past 5 years was referred to a dentist by an anesthesiologist who suspected temporomandibular joint disorder. In oral examination, no specific pathological changes were observed in the oral cavity, including teeth, surrounding alveolar bone, and buccal mucosa. Magnetic resonance imaging and conventional radiography showed no pathological destruction or abnormalities of bone and soft tissue in the temporomandibular joint region. However, pain was precipitated by ordinary daily activities, and the pain alleviating factor was rest. Eventually, the patient was referred to a cardiologist for further evaluation since his pain was induced by physical activity. Coronary artery disease (CAD) was diagnosed using coronary computed tomography angiography, and the pain was considered to be angina pectoris. Percutaneous coronary intervention was successfully done for the patient, after which his orofacial symptoms disappeared. To conclude, isolated craniofacial pain of cardiac origin may lead to patients seeking dental care or visiting orofacial pain clinics. In these settings, dentists and orofacial pain specialists may contribute to the diagnosis of CAD and refer patients for cardiac evaluation and appropriate management.

## 1. Background

During the diagnosis and treatment of orofacial pain, dentists should also consider non-odontogenic etiologies, such as heart diseases, based on pain characteristics, which can be life-threatening. In dental practice, vasovagal syncope and hypertensive crisis are the most common emergencies, accounting for 83.37% and 4.85% of cases, respectively [1]. However, there are also serious life-threatening events that can occur, such as acute coronary syndrome (ACS), anaphylaxis, airway obstruction, and stroke, of which ACS is the most prevalent with 57.14% [1]. The estimated risk of encountering a patient death by British dentists in a 40-year career is reportedly between 1 : 12 and 1 : 19 [2]. According to the Global Burden of Disease, Risk Factors and Injuries study, age-standardized male ischemic heart disease (IHD) mortality per 100,000 individuals in the East Asia region was reported as 61, 83, and 84 in the years 1990, 2005, and 2010, respectively (38% increase) in spite of the overall reduction in IHD mortality worldwide [3].

Coronary artery disease (CAD) refers specifically to the narrowing or blockage of the coronary arteries, whereas IHD is a broader term that encompasses various conditions resulting in inadequate blood supply to the heart muscle, including CAD along with other causes [4]. CAD is typically characterized by substernal pain that may be described as squeezing, heaviness, pressure, weight, vise-like aching, burning, or tightness which radiates to the left arm, shoulder, neck, jaw, and epigastrium [5]. The pain can be precipitated by physical exercise or emotional stress and relieved by rest or nitroglycerin use. Dyspnea and fatigue may also be associated with this condition [6]. Some reports have stated that the pain may be isolated or spread to the orofacial region, especially the jaws and teeth (Table 1) [7–19]. Throat, followed by left mandible, right mandible, left temporomandibular joint (TMJ)/ear region, and teeth are the most commonly reported locations of orofacial pain radiating from heart ischemic diseases [16, 20].

Temporomandibular disorders (TMDs) are a group of musculoskeletal conditions that can cause pain and dysfunction in the TMJ and associated structures. Pain-related TMDs are mainly caused by arthrogenous and myogenous origin pathologies. These types of TMD are complex, chronic pain disorders that involve altered pain sensitivity and increased susceptibility to psychological distress, which can be influenced by both genetic and environmental factors [21]. Pain-related TMD can cause a range of symptoms, including dull aches, sharp pain, clicking or popping sounds in the TMJ, limited mouth opening, and difficulty in chewing or speaking [22]. It is caused by a combination of factors, such as genetics, environment, and psychological factors like stress. Although daytime and nighttime habits and stress can contribute to chronic pain, excessive stress on the TMJ and muscles is not a significant factor. Instead, psychological factors like depression, anxiety, and stress can worsen symptoms and lead to chronic pain [23]. In contrast, orofacial pain as referred pain caused by ACS is usually related to decreased blood flow to the heart muscle, which can radiate to the neck, jaw, and face as well as around the heart [16, 17]. It is usually described as a crushing, pressure-like pain that can be accompanied by sweating, shortness of breath, and other signs of a heart attack.

Patients with orofacial pain referred from cardiac ischemia are likely to seek dental or otolaryngological treatment. Misdiagnosis or delayed diagnosis of such orofacial pain can result in a myriad of problems, ranging from unnecessary dental treatment to life-threatening situations, before receiving appropriate medical care [24]. The suspicion or diagnosis of orofacial pain of cardiac origin by dentists has been uncommonly reported. However, cardiac diseases can cause referred pain in the orofacial region via the vagus nerve, and if not accurately diagnosed, may result in worsening the patient's pain condition. This case report highlights a case of exertional angina that presented as isolated bilateral jaw pain without any cardiac symptoms or medical history.

## 2. Case Presentation

### 2.1. Patient

A 60-year-old male patient was referred to the Orofacial Pain Clinic, Department of Oral Medicine and Oral Diagnosis, Seoul National University Dental Hospital, by an anesthesiologist at the Seoul National University Hospital for the evaluation of suspected TMD for orofacial pain that had persisted for the past 5 years. The patient complained of excruciating bilateral jaw pain of 5-minute durations, which he described as feeling “like the lower jaw was about to fall out.” The pain intensity was reported by the patient as a 10 on the Numerical Rating Scale (NRS). Generally, the NRS ranges from 0 to 10, with 0 being “no pain” and 10 being “the worst pain imaginable”. However, no sleep disturbances were reported. The orofacial symptoms had been present for the preceding 5 years, and the pain had worsened over the last 2 months. The pain was disturbing and exasperating to the point that on two different occasions, he sought treatment in the hospital and also consulted otolaryngology, neurology, and anesthesiology specialists. Pain was first perceived in the preauricular regions, which then radiated down to the masseter muscles. Pain was precipitated by ordinary daily activities including walking and the symptoms usually resolved at rest. However, the patient did not complain of paresthesia, numbness, local fever, or swelling in the orofacial region associated with the pain. During the initial consultation, the patient provided a medical and dental history, which did not reveal any significant findings. He had been a smoker for almost 30 years, was normotensive, and had a body mass index of 25.76 kg/m^2^ (174 cm/78 kg).

### 2.2. Intra/Extraoral Examination and TMJ Imaging

**Figure 1:**
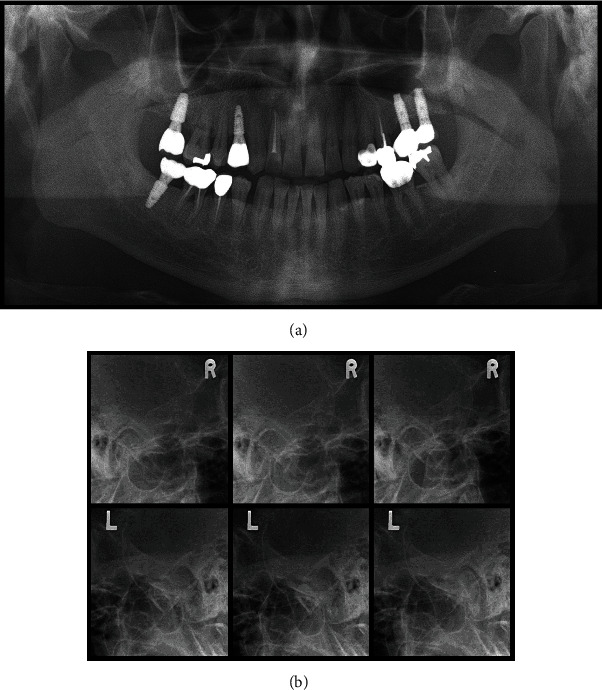
Radiographs of the temporomandibular joints (TMJs). (a) Orthopantomograms of the TMJs showed no significant pathological abnormalities in the temporomandibular joint and jaws. (b) Transcranial view of the TMJs shows no arthritic change in the cortical line of both the joints, and the range of movement of the lower jaw is normal.

In the patient's oral examination, there were no abnormal findings of the oral cavity, including teeth, gums, tongue, and oral mucosal structures. Routine laboratory blood tests, including complete blood cell and differential counts, erythrocyte sedimentation rate, showed normal results. Skull radiological examination, ultrasonography, urine tests, blood tests, and hearing tests performed at other hospitals also provided no plausible explanation for his pain. Extraoral examinations were conducted according to the Research Diagnostic Criteria for Temporomandibular Disorders (RDC/TMD). The maximum mandibular opening recoded was 42 mm. No chin deviation was observed during mouth opening. Upon mandibular movements, no TMJ sounds (such as clicking, wheezing, and popping) were observed, with adequate rotational and gliding movements of the mandible observed. The patient's occlusion was Angle classification I, with a tightly interdigitated bite noted upon application of 8 *μ*m thickness film. On palpating the TMJ and masticatory muscles including temporalis and masseter muscles, familiar pain was not elicited. The patient reported unilateral sleeping and chin-buttressing habits during the investigation as possible contributing factors. The Symptom Checklist-90-Revised was applied for psychological evaluation [25] and yielded normal results. Radiographs, including an orthopantomogram and a transcranial view, revealed no bony abnormalities (Figure 1).

### 2.3. Initial Diagnosis and Magnetic Resonance Imaging

**Figure 2:**
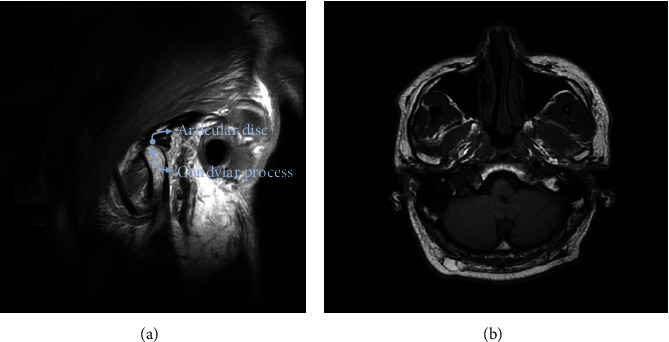
Magnetic resonance imaging (MRI) of the temporomandibular joints (TMJs). (a) T2-weighted sagittal MRI indicates that the anatomical structure of the TMJ region is normal. (b) T2-weighted axial MRI shows no pathologic changes in the TMJ region.

The patient was initially diagnosed with a condition that did not meet the criteria for RDC/TMD and was prescribed self-physical therapy with moist hot packs. In addition, cognitive-behavioral therapy was provided to address behavioral factors that contribute to TMD. Behavior modification was achieved through preventive education, which included recommendations to avoid hard and chewy foods, and to avoid habits like jaw twisting, jaw thrusting, nail biting, and clenching. Clonazepam 0.5 mg orally before bedtime was prescribed. However, the response to these conservative treatments was ineffective. Magnetic resonance imaging (MRI) was performed at the patient's request, which revealed no abnormal findings (Figure 2).

However, the orofacial symptoms still persisted 2 weeks after the MRI. The possibility of cardiac etiology was considered due to lack of local etiology of pain, and since the pain worsened with physical movement. No improvement was observed after typical physical exercise. Following two successive recalls at weekly intervals, he was then referred to the Internal Medicine and Cardiovascular Center of the Seoul National University Hospital 3 weeks subsequent to his initial visit.

### 2.4. Medical Examination, Coronary Computed Tomography, Treadmill Test, and Final Diagnosis

**Figure 3:**
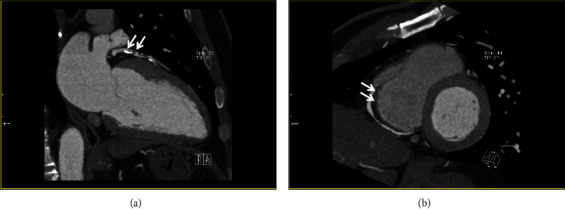
Coronary computed tomography angiography. (a) Left arterial descending artery proximal, mixed plaque with 70–80% stenosis and positive remodeling (white arrows). (b) Right coronary artery (RCA) proximal, mixed plaque with 50–70% stenosis and positive remodeling; RCA mid, mixed plaque with focal near total occlusion; and RCA distal, noncalcified plaque with 40% stenosis (white arrows).

Physical examination revealed a regular pulse of 74 beats per minute and blood pressure of 124/80 mmHg. The remaining physical examination results were unremarkable. Although the patient did not complain of chest pain or dyspnea on exertion, the cardiologist ordered a coronary computed tomography angiography (CCTA) and an exercise treadmill test since the pain was induced by exercise and relieved with rest. CCTA revealed significant CAD affecting all three of his coronary vessels (Figure 3). There was 70–80% stenosis with mixed plaque at the proximal left anterior descending (LAD) artery, near total occlusion or total occlusion of the first diagonal vessel; 50–70% stenosis with mixed plaque at the proximal left circumflex artery (LCx), near total or total occlusion of the distal LCx; and 50–70% stenosis at the proximal right coronary artery (RCA), near total occlusion of the RCA. The exercise treadmill test results were also positive, and the patient was classified as high risk according to the Duke treadmill score.

Further questioning revealed a positive family history of vascular heart disease. Stable angina, also known as angina pectoris, was diagnosed. The patient was admitted, and coronary angiography was performed. There was 70% tubular stenosis at the proximal LAD, near total occlusion at the first diagonal branch and 50% diffused stenosis at the proximal LCx. Moreover, a total occlusion of the distal LCx with Thrombolysis in Myocardial Infarction III flow collaterals from other coronary vessels was observed along with 50% tubular stenosis at the proximal RCA, 70% tubular stenosis, and focal 90% stenosis at the mid-RCA. To evaluate the functional significance of the lesions, fractional flow reserve (FFR) measurements were performed at the LAD and RCA. FFR were 0.75 and 0.62 when hyperemia was induced by adenosine infusion at LAD and RCA, respectively.

### 2.5. Surgical Treatment and Prognosis

The benefits of percutaneous coronary intervention (PCI) as an initial treatment strategy in patients with stable CAD remain controversial [26, 27]. FFR-guided PCI has been reported to improve the outcomes compared to medical therapy alone in patients with stable CAD [28]. PCI was performed, and 3 × 32 mm^2^ and 3 × 26 mm^2^ drug eluting coronary stents were placed at the proximal LAD lesion and mid-RCA lesion, respectively. The procedure was performed successfully, after which all the orofacial symptoms disappeared, confirming that they were caused by CAD.

## 3. Discussion and Conclusions

Unlike previous case reports (Table 1), this study highlights bilateral jaw pain induced by significant CAD, which was resolved after PCI. Pain of cardiac origin is pressure-like and burning in nature, while odontogenic pain is described as pulsatile and sharp, with no sex differences. Bilateral referral pattern of cardiac origin has been reported six times more frequently than unilateral complaint in craniofacial lesions [29].

Orofacial pain of cardiac origin is considered to result from afferent fibers of the vagus nerve that transmit nociceptive stimuli to the cervical neuron cells [30, 31]. In animal studies, noxious electrical and chemical stimulation of cardiac branches of the left vagus nerve reportedly cause the activation of the left spinothalamic tract cells at the level of the trigeminothalamic tract [32, 33]. Based on extensive documentation, both somatic and visceral synapses to the same second-order sensory neurons are found in the outer layers of the dorsal horn [34, 35]. Connections between thoracic and cervical dermatomes (from C2 to T1 roots) and between the cervical dermatomes and the trigeminal nerve are purportedly the cause of referred jaw pain of cardiac origin [18, 19].

One previous study described an association between inferior wall ischemia and facial pain or toothache [36]. Ischemia induces anaerobic metabolism and triggers inflammation, leading to the release of cellular substances such as bradykinin, lactate, proton production (H^+^), adenosine, and substance P [37, 38]. It is believed that one of these substances activates and/or sensitizes the spinal and vagal cardiac afferents, ultimately leading to the sensation of angina and pain referred to the somatic areas [18]. Animal studies using rat models have revealed that ischemia may directly sensitize the afferent nociceptors via a subcategory of capsaicin receptors, the transient potential vanilloid 1 [39].

An uneven distribution of vagal and sympathetic afferent fibers has been observed in different regions of the heart, with a greater concentration of vagal afferent fibers terminating in the inferior-posterior wall of the left ventricle [40, 41]. Experimental evidence from animal studies suggests that the activation of vagal afferent fibers most likely occurs when myocardial ischemia involves the inferior–posterior regions of the left ventricle [42]. However, these proposed mechanisms of referred cardiac pain are insufficient to explain the clinical findings of the present case.

Chest pain or discomfort, whether typical or atypical, is one of the most disconcerting symptoms due to its association with potentially critical heart disease and the risk of death. Ironically, the absence of chest pain is considered a main predisposing factor for delayed or missed diagnoses in acute myocardial infarction [43]. Orofacial pain is reported either independently (6%) or in conjunction with other types of pain (30%) [20]. However, this prospective case study had some limitations. First, the patient was not questioned about his medical history in terms of typical angina symptoms, including pain provoked by exercise. Second, other associated symptoms, such as dyspnea, diaphoresis, fatigue, and vomiting, were not reported and might not have been assessed. Third, patients biased toward anatomic description of pain might have been involved using a diagram [44].

A thorough medical and family history, including relevant symptoms, is crucial in diagnosing craniofacial pain of cardiac origin [45]. Several epidemiologic studies have demonstrated a link between certain risk factors and cardiovascular disease. Causally linked risk factors include tobacco consumption, elevated low-density cholesterol, low high-density cholesterol, high blood pressure, elevated glucose levels, physical inactivity, obesity, and diet. Risk markers that have shown an association are low socioeconomic status, elevated prothrombotic factors (fibrinogen, plasminogen activator inhibitor 1), markers of infection or inflammation, elevated homocysteine levels, elevated lipoproteins, psychological factors (depression, anger proneness, hostility, stress, acute life events), and breakdown in social status (loss of social support and cohesion) [46]. Age, sex, diabetes, hypertension, dyslipidemia, family history of CAD, current smoking status, and symptom type can be easily elicited with careful history taking. In this case report, a full family history that included the history of CAD was identified at the second visit.

Turner et al. recommend that if the signs, symptoms, and investigations of facial pain do not provide a diagnosis, rare causes of facial pain should be considered, such as cardiac ischemia, before diagnosing it as atypical facial pain [9]. It seems plausible that craniofacial pain of cardiac origin is usually accompanied by other symptoms or by a suggestive history such as pain provoked by exercise. The importance of thoroughly assessing a patient's medical history and physical status cannot be overemphasized in the diagnostic process for orofacial pain due to cardiac ischemia. Furthermore, it is necessary to conduct broader studies with a larger sample of patients to determine the characteristics of craniofacial pain of cardiac origin, to avoid unnecessary dental treatments such as tooth extractions and non-indicated TMDs therapies, and to not delay the correct diagnosis of heart disease.

In conclusion, craniofacial pain of cardiac origin may lead to patients seeking dental care. Pain provoked by physical exercise is crucial for the diagnosis, with a thorough medical history for identifying potential differential diagnoses. In these settings, dentists may contribute to the diagnosis of CAD-related orofacial pain and refer patients for cardiovascular evaluation.

## Figures and Tables

**Table 1:** Summary of previous case reports.

Case number [ref], year	Age/sex	Craniofacial pain location (radiation)	Other pain symptoms (radiation)	Precipitating factors	Associated symptom	Medical history, family history	Cardiac treatment	Final diagnosis
1 [7], 1963	62/F	Bilateral Mn, neck, zygoma, temporal area	None	Unknown	Vomit,syncope	HTN	Death of arteriosclerotic heart disease	MI
2 [8], 1981	67/M	Bilateral Mn	None	(+): Walking back to work after lunch	Unknown	Unknown	Propranolol, nitrates	AP
3 [8], 1981	56/F	Bilateral anterior Mx, infraorbital	Neck, shoulder pain	No association with exertion	Dyspnea, orthopnea	Mild scleroderma	Aortocoronary bypass	AP, MI
4 [8], 1981	79/M	Bilateral Mn	Chest pain	(−): Isosorbide dinitrate	Unknown	Unknown	Unknown	MI
5 [48], 1998	56/M	Teeth, Mn	None	(−): Sublingual nitroglycerine	Unknown	Spinal cord injury	CABG	AP
6 [30], 2008	63/F	Left teeth	None	Unknown	Unknown	Grave's disease, chronic depression	Unknown	Referred pain from vagus nerve
7 [49], 2011	48/M	Bilateral TMJ pain	None	Unknown	Unknown	Car accident	CABG	AP
8 [9], 2013	59/F	All teeth, bilateral Mn, Mx	None	(+): Walking,(−): Rest	Nausea	RA, OA, discoid lupus, HTN, IHD family history	Isosorbide mononitrate, statin	AP
9 [10], 1970	72/F	Right Mn	Chest pain on exertion	(+): Effort or stress	Unknown	Unknown	Unknown	AP
				(−): Rest and vasodilator				
10 [11], 1975	32/M	Left Mx posterior teeth	Chest pain (shoulder, back pain)	(+): Physical activities	Unknown	None	CABG	AP, MI
				(−): Nitroglycerine				
11 [12], 2005	50/F	Bilateral Mn, Mx, left temporal area	Chest pain (neck, arm pain)	(+): Exercise emotion	Unknown	DM, CABG	Unknown	AP
				(−): Relax				
12 [13], 2006	65/F	Bilateral zygoma, (Mn and left temporal region)	Chest pain, arm pain (neck pain)	(+): Mandibular movement, walking, climbing stairs	Nausea, diaphoresis, vomiting	Thyroidectomy, MI	Propranolol, isosorbide dinitrate, aspirin	MI, AP
13 [14], 2006	61/F	Bilateral Mx, Mn (eye)	Chest pain	Unknown	Unknown	DM, HTN, smoking hypothyroidism	Stent in left coronary artery	AP
14 [50], 2010	80/M	The area of dental extraction (forehead and neck)	Chest pain	(+): Walking	Unknown	DM, HTN	Angioplasty of the circumflex artery with implantation of a sirolimus eluting stent	AP
15 [16], 2012	54/M	Third quadrant including teeth	Chest, neck, and shoulder pain	Unknown	Unknown	NSAIDs (neck and back pain), HTN, type II DM, smoking (15 cigarettes/day), moderate drinker	Medication	Unstable angina
16 [16], 2012	79/M	Left Mn	Left arm	Unknown	Unknown	Type II DM, left knee prosthesis	Unknown	IHD
17 [17], 2015	Late 50s/F	Left face and Mx	None	Percussion and palpation on periradicular area	Unknown	Unknown	Unknown	MI and IHD

Mn, mandible; Mx, maxilla; HTN, hypertension; DM, diabetes mellitus; RA, rheumatoid arthritis; OA, osteoarthritis; MI, myocardial infarction; AP, angina pectoralis; IHD, ischemic heart disease; Ref, reference; CABG, coronary artery bypass graft; (+), aggravating factor; (−), relieving factor.

## Data Availability

The data used to support the findings of this study are available from the corresponding author upon request.

## Abbreviations

TMJ: Temporomandibular joint
CAD: Coronary artery disease
CCTA: Coronary computed tomography angiography
PCI: Percutaneous coronary intervention
ACS: Acute coronary syndrome
IHD: Ischemic heart disease
TMD: Temporomandibular disorder
RDC/TMD: Research diagnostic criteria for temporomandibular disorders
MRI: Magnetic resonance imaging
CT: Computed tomography
LAD: Left anterior descending artery
LCx: Left circumflex artery
RCA: Right coronary artery
FFR: Fractional flow reserve.
